# Improved Working Memory Performance through Self-Regulation of Dorsal Lateral Prefrontal Cortex Activation Using Real-Time fMRI

**DOI:** 10.1371/journal.pone.0073735

**Published:** 2013-08-27

**Authors:** Gaoyan Zhang, Li Yao, Hang Zhang, Zhiying Long, Xiaojie Zhao

**Affiliations:** 1 State Key Laboratory of Cognitive Neuroscience and Learning, Beijing Normal University, Beijing, China; 2 College of Information Science and Technology, Beijing Normal University, Beijing, China; Yale University, United States of America

## Abstract

Working memory is important for a wide range of high-level cognitive activities. Previous studies have shown that the dorsal lateral prefrontal cortex (DLPFC) plays a critical role in working memory and that behavioral training of working memory can alter the activity of DLPFC. However, it is unclear whether the activation in the DLPFC can be self-regulated and whether any self-regulation can affect working memory behavior. The recently emerged real-time functional magnetic resonance imaging (rtfMRI) technique enables the individuals to acquire self-control of localized brain activation, potentially inducing desirable behavioral changes. In the present study, we employed the rtfMRI technique to train subjects to up-regulate the activation in the left DLPFC, which is linked to verbal working memory. After two rtfMRI training sessions, activation in the left DLPFC was significantly increased, whereas the control group that received sham feedback did not show any increase in DLPFC activation. Pre- and post-training behavioral tests indicated that performance of the digit span and letter memory task was significantly improved in the experimental group. Between-group comparison of behavioral changes showed that the increase of digit span in the experimental group was significantly greater than that in the control group. These findings provide preliminary evidence that working memory performance can be improved through learned regulation of activation in associated brain regions using rtfMRI.

## Introduction

Working memory involves the temporary storage and manipulation of information that is assumed to be necessary for a wide range of complex cognitive activities such as reasoning, comprehension and learning [1]. According to a multi-component model proposed by Baddeley and Hitch, working memory could be divided into an attention control system, the central executive, and two modality-based temporary storage systems, the phonological loop and the visuospatial sketch pad. Among the three systems, the central executive system controls attention and information flows to and from the phonological loop and visuospatial sketch pad, which separately buffer the verbal-acoustic and visual material [2].

The functional brain anatomy underlying the three systems was explored by neuroimaging studies. It was suggested that the phonological loop primarily recruited regions in the left hemisphere, including the temporoparietal region and Broca’s area; the visuospatial sketch pad predominately engaged regions in the right hemisphere, including the frontoparietal cortex and the occipital cortex [3,4]; and the central executive function was mainly mediated by the prefrontal cortex [5,6]. With respect to the central executive function, accumulating evidence showed that the dorsal lateral prefrontal cortex (DLPFC) played a crucial role in a variety of executive control processes. Funahashi et al. used a spatial working memory task to explore the prefrontal cortex of monkeys and the result of single-unit recording showed that the DLPFC controlled information maintenance [7]. By examining the activation of the human prefrontal cortex as verbal and spatial working memory tasks were performed simultaneously, D’ Esposito et al. identified the key role of the DLPFC in coordinating two concurrent tasks [8]. To further determine the functional importance of the DLPFC in working memory, Barbey et al. studied patients with damage in the DLPFC and observed a deficit in the manipulation of verbal and spatial information [9]. These findings imply that the DLPFC is a key node that supports working memory.

When exploring the relationship between the DLPFC and working memory performance, it was shown that behavioral training of working memory can alter the activity of DLPFC. Jansma et al. reported that the activation of the left DLPFC was decreased after consistent practice of a verbal Sternberg task, and the response to this task was faster and more accurate [10]. Through training of visuospatial working memory task, Olesen et al. found that activation in the right DLPFC was increased and the response time for the trained task decreased [11]. By contrast, to determine the functional contribution of DLPFC to a behavior, various brain stimulation approaches that can temporarily alter the irritability of a local cortical region have been used to supplement the behavioral study. One study used the repetitive transcranial magnetic stimulation (rTMS) to inhibit the cortical excitability of the left DLPFC, and found that performance of the random number generation task was disrupted [12]. Other studies showed that anodal stimulation over the left DLPFC using transcranial direct current stimulation (tDCS) improved performance of the digit span task [13] and the alphabetical 3-back task [14]. These studies demonstrate that the activity of DLPFC can be altered by behavioral training and that the working memory behaviors can also be modulated by the activity intensity of the DLPFC.

The recently emerged real-time functional magnetic resonance imaging (rtfMRI) technique provides a new approach to mediate behavioral performance by regulating brain activation [15]. In contrast to the brain stimulation method, the rtfMRI technique locates a region of interest (ROI) with high spatial resolution and provides the blood oxygenation level-dependent (BOLD) signal in the ROI as feedback to guide individuals to self-regulate brain activation, consequently inducing desirable behavioral changes [16]. The existing rtfMRI studies have shown that individuals can learn to regulate brain activation in some localized regions, such as rostral anterior cingulate cortex (rACC) [17], primary motor cortex [18], inferior frontal gyrus (IFG) [19], auditory cortex [20], anterior insular [21,22], ventral premotor area (PMA) [23], amygdala [24] and rostrolateral prefrontal cortex [25]. Using the rtfMRI technique, researchers also examined the behavioral effects that depend on the self-regulation of local brain activation. deCharms et al. found that successfully regulating the activation of the rACC led to decreases in the ongoing level of chronic pain [17]. Caria et al. observed that acquired control over activation in the left anterior insular enhanced the perception of visual emotional stimuli [21]. Sitaram et al. reported that intentionally increasing the activation in the ventral PMA improved performance of a visuomotor tracking task [23]. However, few studies have used rtfMRI to investigate the regulation of BOLD activity in regions related to working memory and the triggered behavioral effect by the self-regulation.

Because the DLPFC is regarded as a key node in working memory and the left DLPFC is mostly related to the performance of verbal working memory [13,26], the present study used the left DLPFC as the target ROI for rtfMRI training and chose the verbal working memory task to evaluate the effect of training. We aimed to explore whether activation in the DLPFC can be self-regulated through rtfMRI training and, if so, whether successful up-regulation of activation in the DLPFC can lead to an improvement in working memory behavior.

## Materials and Methods

### Human subjects

A total of thirty healthy, right-handed college students participated in the study. Eight male and seven female subjects (mean age: 21.47±3.83 years) were randomly assigned to the experimental group. The other fifteen subjects, including eight males and seven females (mean age: 21.87±3.41 years), constituted the control group. There was no significant difference in age between the two groups (*p*=0.57). The subjects had no history of psychiatric or neurological disorders and had not previously participated in memory training or instrumental learning.

All subjects signed the informed consent before the scans were conducted. The experiment was approved by the Institutional Review Board (IRB) of the State Key Laboratory of Cognitive Neuroscience and Learning in Beijing Normal University.

### Experimental procedure

**Figure 1:**
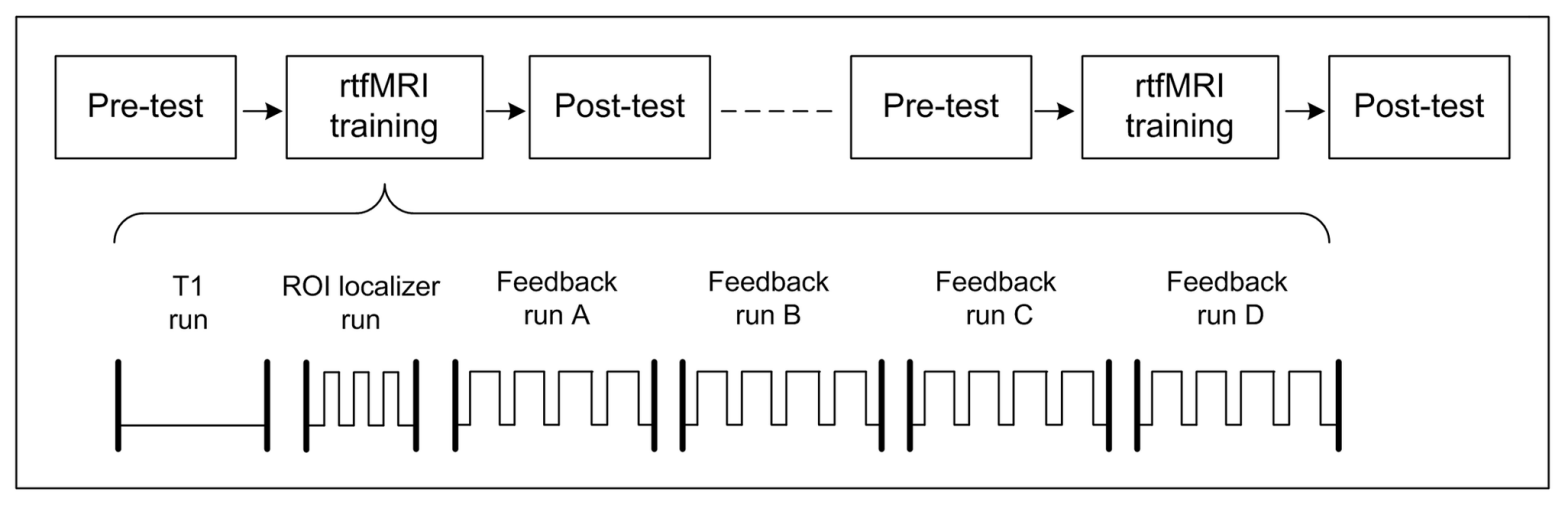
Outline of the whole experimental procedure. Each rtfMRI training session comprised six runs. T1 run was a 10-minute T1-weighted scan. The ROI localizer run comprised four digital 0-back blocks alternated with three digital 3-back blocks; each n-back block lasted for 34.0 s, with 4.0 s for the cue and 30.0 s for the tasks. The feedback run lasted for 6.5 minutes and included four up-regulation blocks (60.0 s each) separated by five baseline blocks (30.0 s each), with the beginning of a baseline block.

The whole experimental procedure included two rtfMRI training sessions separated by seven days and the pre- and post-test using behavioral tasks respectively performed on the day before and after each rtfMRI training session (Figure 1).

For each rtfMRI training session, a T1-weighted image was firstly scanned for the overlay of functional map. Then, a digital 3-back task was performed in the ROI localizer run. According to the individual statistical result of the digital 3-back task, a rectangular area (5×4 voxels) centered on the local activation maximum of the left DLPFC was selected as the target ROI. To cancel out the unspecific global effects, a control ROI was defined as a task-unrelated area (square area, 6×6 voxels) in the right hemisphere of the same slice. In the following feedback runs, the feedback signal was calculated as the difference of BOLD signal changes between the two ROIs according to the equation (BOLD_regulation_-BOLD_baseline_)_targetROI_ - (BOLD_regulation_-BOLD_baseline_)_controlROI_ in which the term BOLD_regulation_ referred to the signal in the current time point of the regulation block and the term BOLD_baseline_ represented the average signal of the preceding rest block. During the regulation blocks, the feedback signal was transformed into visual feedback of graduated thermometer with an increasing or decreasing number of bars once per repetition time (TR). Because the left DLPFC is significantly activated in the self-ordered task [27] and in the backward reciting digit/letter sequence task [28,29], subjects were instructed to use a cognitive strategy of backward reciting the self-generated sequences sub-vocally to increase the number of bars in the thermometer. The content, length, and difficulty of the sequences they generated and the speed of recitation could be adjusted according to the feedback. The aim was to persistently increase the number of bars in the thermometer as much as possible. During the baseline blocks, ‘+’ was presented on the screen, and subjects were instructed to rest and not recall anything about the regulation. In the whole feedback run, subjects were asked to relax and maintain uniform breathing and heartbeat. To verify the training effect, subjects in the control group completed the same experimental procedure and received the same instructions, except that they were provided with a sham feedback signal in the feedback runs. The sham feedback was randomly chosen from the feedback signals of five subjects in the experimental group whose self-regulation results were in an intermediate level of all the subjects. After the scanning, subjects were asked to complete a questionnaire to record the detailed strategies they used and any discomfort they experienced during the scanning.

For the pre-/post-test, three different types of behavioral tasks, including the criterion, near transfer and far transfer tasks were completed. The digit span task, which measures the short-term storage and manipulation of verbal information [29], was selected as the criterion task to conform with the regulation process. This task included forward and backward digit span according to the Wechsler Adult Intelligence Scale revised in China [30]. The letter memory task, which tests the maintenance and manipulation especially the updating of verbal information [31], was used to evaluate the near transfer effect. The test involved 10 lists of letter sequence with varied length from 6–15 letters; when a list ended, the subjects were asked to enter the last 4 letters using the keyboard within a time limit of 6.0 s. The spatial 3-back and Stroop color-word tasks were used as the far transfer tasks to separately assess the monitoring of visuospatial information [32] and the inhibition of conflict [33]. The spatial 3-back task included 6 lists, and each lasted for 30.0 s. The Stroop color-word test consisted of 72 randomly presented Chinese characters with 24 congruent, 24 incongruent and 24 neutral stimuli; the total duration was 144s. For the letter memory, spatial 3-back and Stroop color-word tasks, stimulus presentation and response collection were carried out using E-prime 1.1 software [34]. All the behavioral tasks were designed into four counterbalanced sets.

### rtfMRI data acquisition and online analysis

Brain images were acquired using a SIEMENS 3.0 T scanner at the MRI Center of Beijing Normal University. For each subject, a T1-weighted magnetization-prepared rapid gradient-echo (MPRAGE) sequence was used to obtain the anatomical images (matrix = 256×256, 176 partitions, 1 mm^3^ isotropic voxels, TR = 2530 ms, echo time (TE) = 3.45 ms, flip angle= 7°). A single-shot T2*-weighted gradient-echo, echo-planar image (EPI) sequence was used for the functional imaging acquisition (TR = 2000 ms, TE = 30 ms, matrix = 64×64, In-plane resolution = 3.125×3.125 mm^2^, slice = 33, slice thickness = 4.0 mm, slice gap = 0.6 mm, flip angle = 90°). To reduce movement, two foam cushions were used to immobilize the subjects’ head.

The online analysis of whole-brain fMRI data in the ROI localizer run and feedback runs was performed with Turbo-Brain Voyager software (Brain Innovation, Maastricht, Netherlands). The data analysis included online incremental 3D motion correction, drift removal, spatial smooth (full width at half maximum (FWHM) = 8mm) and incremental statistical analysis based on the general linear model (GLM). The threshold for statistical significance was set at *p*<0.001, with a minimum cluster size of 20 contiguous significant voxels. The statistical map was updated once per TR and was presented to the experimenter for reference in the interface of Turbo-Brain Voyager software.

### Offline data analysis

The fMRI data from the ROI localizer run was analyzed with the SPM8 software package (http://www.fil.ion.ucl.ac.uk/spm/). Before preprocessing, the first four volumes were excluded to account for T1 equilibration effects. The remaining EPI volumes were corrected for head motion, normalized to the Montreal Neurological Institute (MNI) space, resliced into a resolution of 3×3×4 mm^3^ voxels and spatially smoothed using a Gaussian kernel with FWHM of 8 mm. After preprocessing, data from each subject were high-pass filtered, and then GLM analysis was applied to compute an individual statistical map. A one-sample *t*-test was performed to obtain the group activation map. The number of correct hits and the associated reaction time of the digital 3-back task in the ROI localizer run were also calculated to evaluate the activation-behavior relationship.

To precisely assess the self-regulation of activation in the left DLPFC at the group level, we defined an offline ROI, slightly different from the feedback ROI, with an advantage of using the Brodmann’s Area (BA) template for reference. The offline ROI was defined as a spherical region with a radius of 6 mm and centered on the peak value of the left DLPFC in BA 9 according to the group statistical results of ROI localizer run. The self-regulation effect was preliminarily evaluated by testing whether the percent signal changes in the ROI increased across runs using linear regression analysis. To further examine the differences of percent signal changes between runs/groups, two-way repeated-measures ANOVA with run (eight runs; within-subjects) and group (two groups; between-subjects) as the main factors was performed using SPSS 13.0 (SPSS Inc., Chicago, IL, USA).

The same individual analysis as the ROI localizer run was performed for the feedback runs. Regions recruited during the regulation were identified using a one-sample *t*-test. A two-sample *t*-test was employed run by run to identify regions showing stronger activation in the experimental group than the control group. The statistical threshold for these analyses was set at *p*< 0.001, with a minimum cluster size of 20 contiguous significant voxels.

For the behavioral tasks, the digit span including forward and backward span, number of correct responses in the letter memory task, number of correct hits and the associated reaction time in the spatial 3-back task, and the reaction time in the Stroop color-word test were separately calculated for each subject. Paired *t*-tests between the behavioral tests after the second rtfMRI training session (2nd post-test) and that before the first rtfMRI training session (1st pre-test) were conducted in each group to assess any improvement in behavior after the training. Moreover, further comparison of behavioral changes from 1st pre-test to 2nd post-test in the experimental group with that in the control group was conducted to precisely evaluate the behavioral improvement induced by the rtfMRI training.

## Results

### ROI localizer

**Figure 2:**
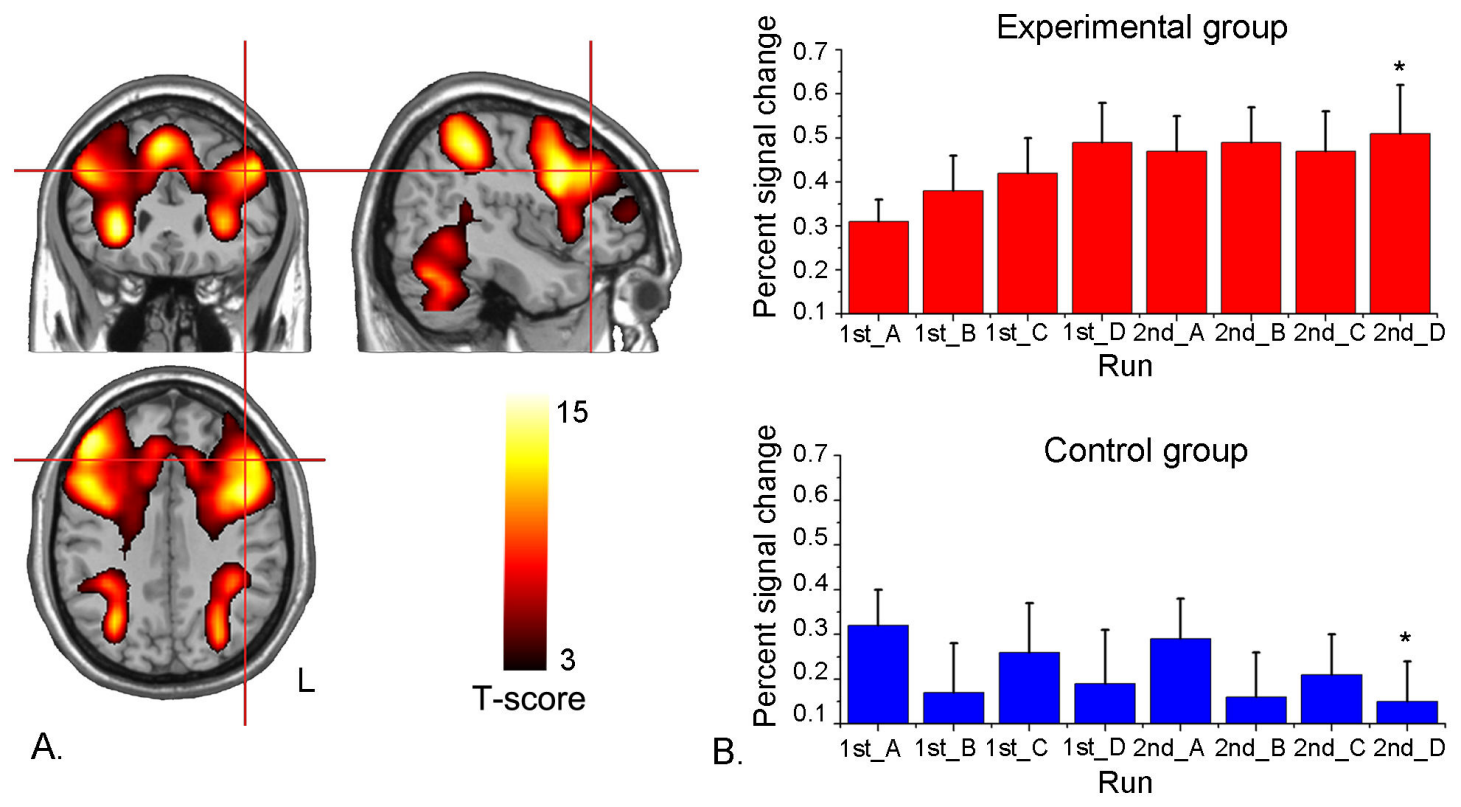
ROI localizer and analysis. A. Group activation map of the ROI localizer run (*p*<0.001, cluster > 20). The cross refers to the maximum activation in the ROI of the left DLPFC (BA9) in MNI coordinate (-45, 29, 34). The left is on the reader’s right. B. The percent signal change of the ROI in the experimental group and the control group during eight feedback runs (Run 1st_A to 2nd_D respectively represent the feedback run A to D in the first and second rtfMRI training session). Error bar means the standard error. *: significant difference in the comparison of run 2nd_D with run 1st_A (*p*<0.05).

One-sample *t*-tests on the ROI localizer run data for the two groups together showed that the digital 3-back task significantly activated the bilateral DLPFC, supplement motor area (SMA), bilateral PMA, left IFG, bilateral posterior parietal cortex (PPC), bilateral insular, caudate, putamen, thalamus and cerebellum. The peak value in the DLPFC, in BA 9, was located at the MNI coordinates x=-45, y=29, z=34 (Figure 2A). Moreover, the activation in the left DLPFC ROI was positively correlated with the number of correct hits (*r*=0.51, *p*<0.05, *N*=15) and negatively correlated with the reaction time of the correct hits (*r*=-0.49, *p*<0.05, *N*=15).

### ROI analysis of percent signal changes

The group averaged percent signal changes of the left DLPFC in the feedback runs is illustrated in Figure 2B. Linear regression analysis showed a progressively increase of the mean percent signal changes in the left DLPFC in the experimental group (y = 0.025x + 0.333, *R* ^2^=0.75, *p*<0.01). In comparison, no obvious regulation trend was observed for the mean percent signal changes in the control group (y = -0.015x + 0.282, *R* ^2^=0.31, *p*=0.15). The repeated-measure ANOVA (main factors: group and run) revealed that there was a marginal significant main effect of group (*F*(1,28) = 4.089, *p*=0.053) and a significant interactive effect between group and run (*F*(7,196) =2.795, *p*<0.05). No significant effect of run was observed (*F*(7,196)=0.671, *p*=0.616). Pair-wise comparison of the last feedback run (run 2nd_D) with the first feedback run (run 1st_A) suggested a significant increase of percent signal changes in experimental group but a significant decrease of percent signal changes in the control group (Figure 2B). Comparison of percent signal changes between the two groups showed no significant difference in run 1st_A (*p*=0.46) but a notable difference in run 2nd_D (*p*<0.05).

### Whole-brain activation analysis

**Figure 3:**
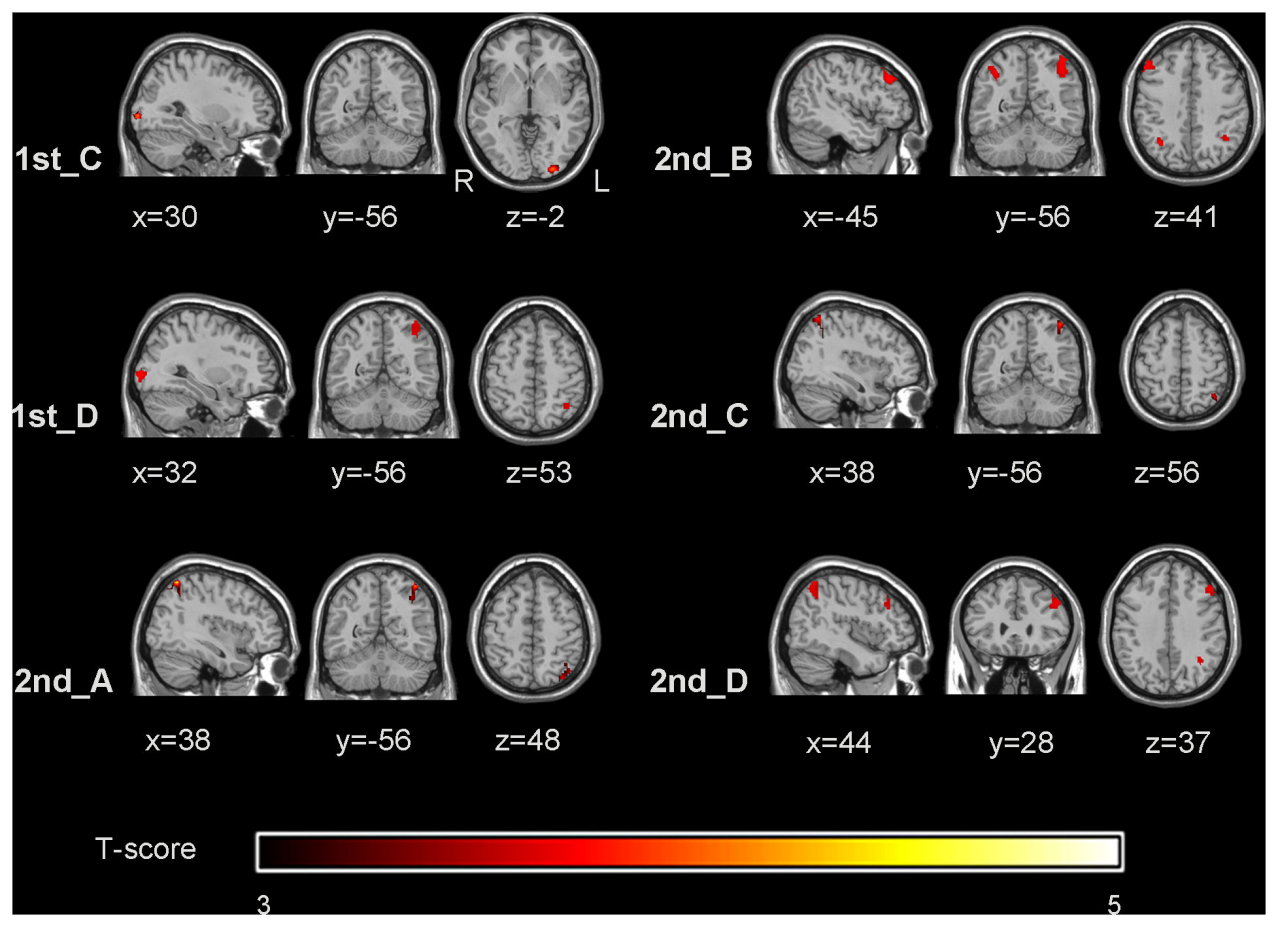
Group differences during each feedback run. Brain regions showing stronger activation in the experimental group than in the control group (two-sample *t*-test, *p*<0.001, cluster > 20) were observed in run 1st_C to run 2nd_D, but not in run 1st _A and run1st_B. The left is on the reader’s right.

Whole-brain analysis of the feedback runs showed significant activations in the bilateral DLPFC, PMA, SMA, ACC, left IFG, bilateral PPC, bilateral insular, caudate, putamen, thalamus, occipital lobe and cerebellum. Between-group comparison indicated stronger activation in bilateral DLPFC, PPC and left middle occipital gyrus (MOG) in the experimental group compared with the control group (Figure 3).

### Pre- and Post-test of behavioral tasks

**Figure 4:**
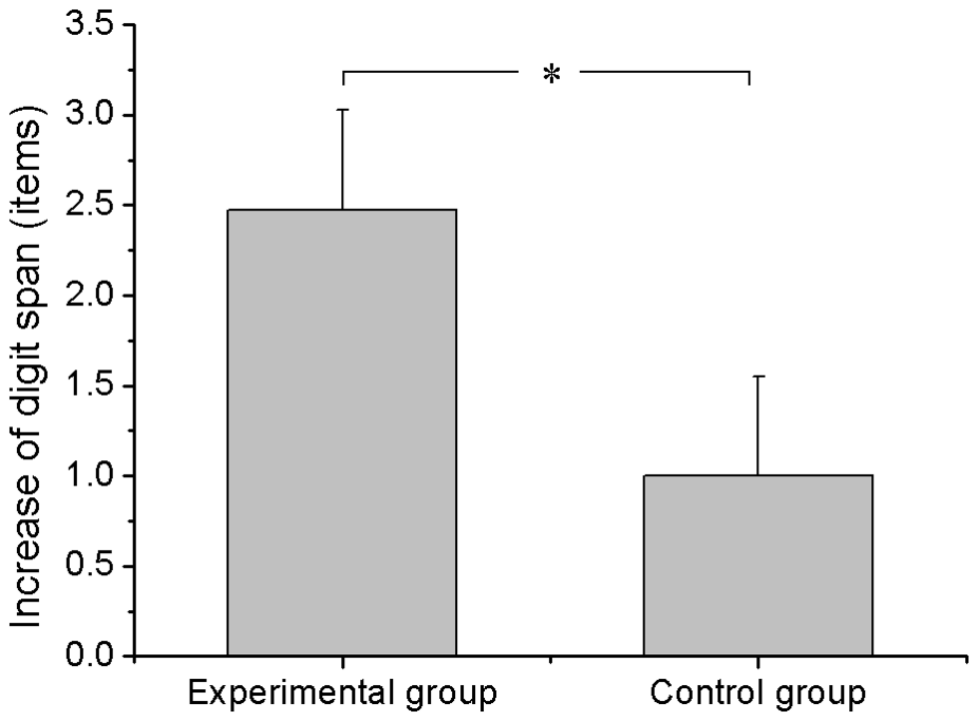
Behavioral changes in the digit span task. The increase of digit span from 1st pre-test to 2nd post-test was significantly greater in the experimental group than that in the control group. Error bar means the standard error. *: *p*<0.05.

The behavioral comparison of 1st pre-test with 2nd post-test in each group and the comparison of behavioral changes between the two groups were displayed in Table 1. Although the digit span was increased in both groups after the rtfMRI training, between-group comparison of behavioral changes indicated that the increase of digit span in the experimental group was significantly greater than that in the control group (Figure 4). For the letter memory task, comparison of correct responses in 1st pre-test with that in 2nd post-test demonstrated a significant enhancement in the experimental group but not in the control group, and no significant difference of behavioral changes was observed between groups (Table 1). For the spatial 3-back task and the Stroop color-word task, performance of the two tasks was significant improvement from 1st pre-test to 2nd post-test in both groups and no significant difference of behavioral changes was found between the two groups (Table 1).

**Table 1:** Behavioral performance in the experimental group and the control group before and after the rtfMRI training.

	**1st pre-test Mean (S.E.)**	**2nd post-test Mean (S.E.)**	**2nd post vs. 1st pre**	**Group difference^a^**
**Digit span (items)**				
Experimental group	17.73 (0.65)	20.13 (0.91)	*p*=0.001*	*p*=0.047*
Control group	17.00 (0.81)	18.00 (0.60)	*p*=0.046*	
**Letter memory (items)**				
Experimental group	5.93 (0.68)	7.53 (0.46)	*p*=0.009*	*p*=0.250
Control group	6.60 (0.58)	7.60 (0.47)	*p*=0.070	
**Spatial 3-back correct hits (items)**				
Experimental group	13.33 (0.74)	16.67 (0.94)	*p*=0.002*	*p*=0.305
Control group	11.53 (1.26)	15.67 (1.09)	*p*=0.002*	
**Spatial 3-back reaction time (ms)**				
Experimental group	781.12 (47.06)	588.40 (57.38)	*p*=0.001*	*p*=0.487
Control group	797.36 (41.80)	606.85 (44.55)	*p*=0.000*	
**Stroop reaction time (ms)**				
Experimental group	635.76 (23.12)	563.19 (16.83)	*p*=0.000*	*p*=0.261
Control group	641.96 (32.42)	583.24 (30.55)	*p*=0.000*	

S.E.-standard error.

^a^ Comparison of behavioral changes from 1st pre-test to 2nd post-test in the experimental group with that in the control group.

* *p*<0.05

## Discussion

The present study used rtfMRI to investigate the self-regulation of brain activation in the left DLPFC and the modulation of working memory behaviors. The results showed that activation in the left DLPFC was significantly up-regulated and the performance of verbal working memory was improved after rtfMRI training. These findings indicated that the brain’s high-level cognitive behavior could be promoted through learned regulation of brain activation in associated areas using rtfMRI.

In the ROI localizer run, activation of the ROI in the left DLPFC showed a significantly positive correlation with the number of correct hits in the digital 3-back task and a notably negative correlation with the associated reaction time. This result was consistent with a previous study showing that activation in the left DLPFC was correlated with working memory performance [35] and indicated that selecting the left DLPFC as the target ROI for rtfMRI training was reasonable. Moreover, the improved verbal working memory performance (Figure 4) observed in the present study after up-regulation of left DLPFC activation also verified the importance of the DLPFC in working memory.

ROI analysis of the percent signal changes in the left DLPFC during the feedback runs (Figure 2B) revealed no significant difference of percent signal changes in feedback run 1st_A between the two groups. With the training going on, significant increase of percent signal changes was observed in the experimental group but not in the control group. The notable between-group difference suggested that the feedback information played a critical role in the regulation process and demonstrated that activation in the left DLPFC can be up-regulated through rtfMRI training. In the post-experiment questionnaire, most subjects reported that up-regulation of activation in the target ROI was achieved by increasing the difficulty and randomness of the generated sequences. This is consistent with the previous findings that signal change in the left DLPFC was correlated with working memory load [36] and that random sequence generation that engaged the central executive component of working memory was associated with activation in the left DLPFC [37]. It should be noted that the increase of percent signal change in the target ROI was first sharp and then mild. A previous study indicated that activation in the DLPFC followed an ‘inverted-U’ shape as working memory load increased [38]. The subjects in our study also reported that if the sequence they generated was too difficult to recite backward, the bars of the thermometer decreased; thus, to avoid decrease, they did not select sequences that were too difficult. Moreover, in a study of computerized training of working memory, activation of the DLPFC also increased rapidly at first and then remained nearly constant (Figure 3d in [11]). According to Desimone’s hypothesis [39], the observed regulation effect may be due to two parallel mechanisms: an enhancement mechanism for active working memory that caused the initial rapid increase and a repetition suppression mechanism that was engaged automatically by continuous feedback training.

Whole-brain analysis of the feedback runs revealed that the activated regions were generally consistent with the regions reported in the previous backward recitation studies [28,29,40]. Although the between-group comparison showed that there was no difference of activations in the bilateral DLPFC, PPC and left MOG in run 1st_A, as the training progressed, stronger activations in these regions appeared in the experimental group (Figure 3). Evidences from neuroimaging studies indicated that the DLPFC was primarily engaged in executive functions, such as information manipulation in the digit span task [29], and the PPC was part of the phonological loop, mediating the storage of verbal information [41]. Kosslyn et al. proposed that activation of the occipital area in verbal working memory was related to visual imagery [42]. The stronger activations of the DLPFC, PPC and MOG in the experimental group may reflect the more recruitment of these regions to support for the learning of up-regulating the activation in the target ROI.

By means of the rtfMRI training, the improvement of performance in the criterion task (digit span) was significantly greater in the experimental group than that in the control group (Figure 4). This result provided evidence that learned regulation of activation in the DLPFC can lead to improvement in working memory behavior. For the near transfer task (letter memory), although the between-group comparison of behavioral changes was not significant, notable improvement of behavior from 1st pre-test to 2nd post-test was observed in the experimental group but not in the control group (Table 1). This improvement may be induced by the weak near transfer effect or by other factors such as the inter-individual difference. Because there were few studies that examined the near transfer effect of a short-term (less than 2 hours) working memory training [43], further studies are needed to explore this phenomenon. For the two remaining far transfer tasks, the spatial 3-back and Stroop color-word tasks, obvious within-group enhancements were detected in both groups and no significant between-group differences of behavioral changes were found in either of the tasks (Table 1). Previous studies reported that a far transfer effect was usually observed after approximately 10 hours working memory training [44,45]. It is very possible that the short-term rtfMRI training did not have an effect on the far transfer task.

Overall, the present study demonstrated that up-regulation of DLPFC activation by rtfMRI training may improve working memory. Namely, the training approach based on rtfMRI technique may facilitate the augment of memory ability and the rehabilitation of memory function. Clinically, working memory impairment is a major feature of many neurologic and psychiatric disorders, such as attention deficit hyperactivity disorder (ADHD) [46–48], Alzheimer’s disease (AD) [49,50] and schizophrenia [51,52]. Neuroimaging studies have indicated that the dysfunction in prefrontal cortex may be associated with working memory impairment [52–54]. Therefore, improving the performance of working memory by self-regulating the cortical activities in the prefrontal areas through rtfMRI training appears to be promising for clinical application.
